# COVID-19 masquerading as a non-convulsive status epilepticus

**DOI:** 10.1186/s12245-022-00412-w

**Published:** 2022-01-21

**Authors:** Sofie Moorthamers, Thierry Preseau, Saïd Sanoussi, Marie-Dominique Gazagnes

**Affiliations:** 1 Emergency Department, Brugmann University Hospital, Université Libre de Bruxelles, Brussels, Belgium; 2 Radiology Department, Brugmann University Hospital, Université Libre de Bruxelles, Brussels, Belgium; 3 Stroke and Neurological Rehabilitation Department, Brugmann University Hospital, Université Libre de Bruxelles, Brussels, Belgium

**Keywords:** Non-convulsive status epilepticus, COVID-19 atypical ED presentations, Reversible clinical picture of SARS-CoV-2 infection, EEG availability at ED

## Abstract

Since the outbreak of the coronavirus disease 2019 (COVID-19) pandemic, caused by the severe acute respiratory syndrome coronavirus 2 (SARS-CoV-2), more and more atypical presentations of COVID-19 are being reported. Here, we present and discuss non-convulsive status epilepticus (NCSE) as presenting symptom of SARS-CoV-2 infection at the Emergency Department.

## Introduction

The outbreak of SARS-CoV-2 in Wuhan, Central China, in January 2020, has resulted in a global pandemic with significant morbidity and mortality [1]. At the beginning of the pandemic, COVID-19 was primarily considered a pulmonary disease; however, the clinical spectrum of SARS-CoV-2 infection is very broad and extrapulmonary involvement is increasingly cited in literature. Various neurological presentations have been linked to SARS-CoV-2, but only a few recent reports have described non-convulsive status epilepticus (NCSE) as the first manifestation of SARS-CoV-2 infection [2–4]. Especially when seen in conjunction with other underlying diseases, NCSE often goes unrecognized and NCSE is commonly underdiagnosed in the Emergency Department (ED) and intensive care unit (ICU). However, failure and delay to diagnose NCSE may cause inappropriate treatment and irreversible brain damage [5]. Therefore, it is critically important for the emergency physician to consider NCSE as a—if treated in a timely manner—completely reversible neurological picture of COVID-19 disease. In order to bring further awareness of NCSE as a rare initial manifestation of COVID-19 disease, we here present a case of a SARS-CoV-2-associated NCSE in a 76-year-old-female presenting with unexplained coma at the ED.

### Narrative/case presentation

**Fig. 1:**
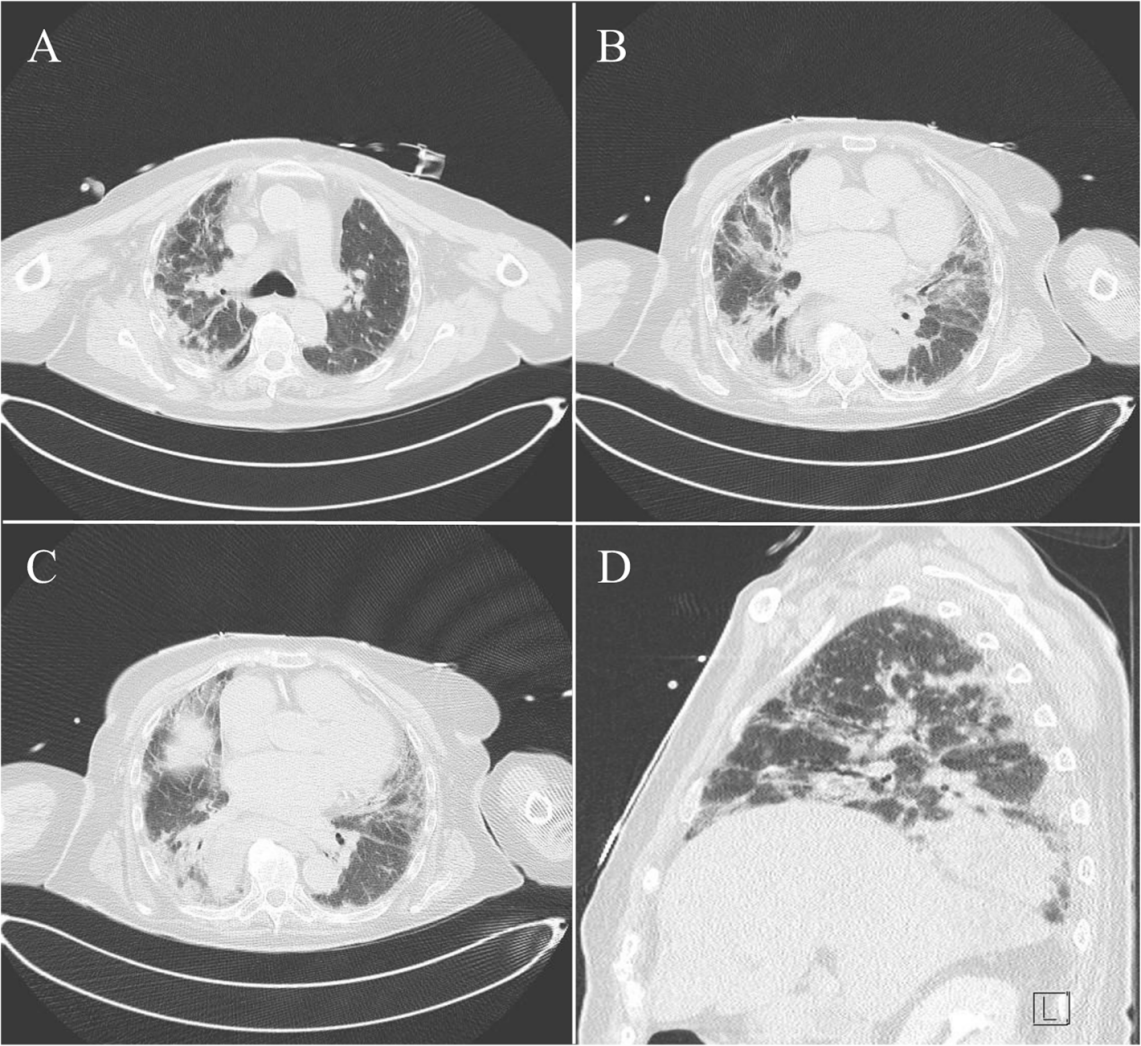
Patient’s chest CT showing ground glass opacities, predominantly peripheral, involving the five lung lobes, compatible with severe COVID-19 pneumonia. **A**–**C** Axial view. **D** Sagittal view

**Fig. 2:**
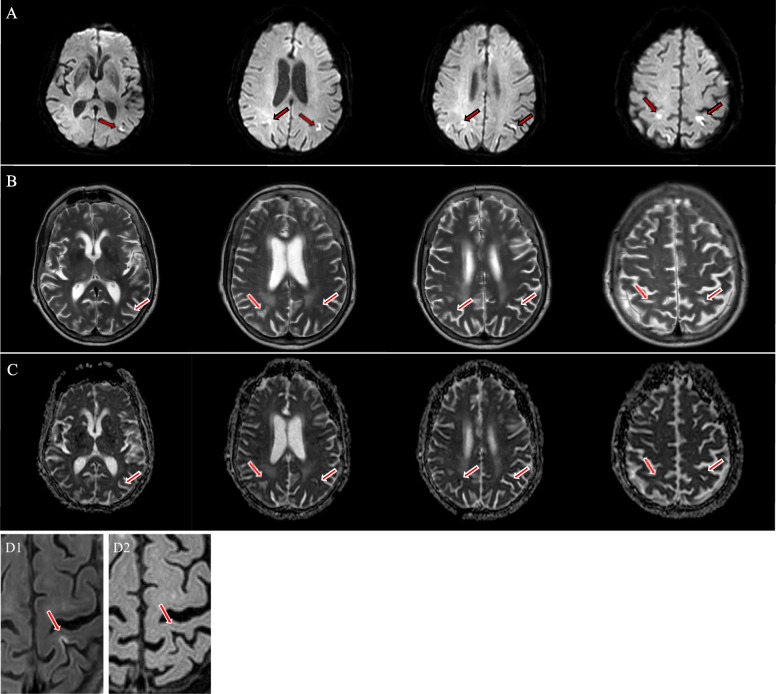
Initial brain MRI: arrows show the focal cortical signal hyperintensity changes on diffusion weighted (**A**) and T2 weighted images (**B**) with reduced apparent diffusion coefficient on ADC map (**C**). Lesions are located in the cortical gray matter and lack arterial distribution. They are topographically compatible with transient postictal MRI changes indicating the presence of cytotoxic and vasogenic edema. Follow-up MRI revealed complete resolution of signal changes and no new other lesions. **D** FLAIR signal hyperintensity on initial brain MRI (D1) and normalized FLAIR signal intensity on follow-up MRI illustrating the reversibility of postictal MRI abnormalities (D2)

A 76-year-old female was brought to the Emergency Department (ED) after being found unconscious at home with a recorded room air saturation of 86%. She had a past medical history significant for arterial hypertension, hypercholesterolemia, transient ischemic attack, and non-valvular atrial fibrillation. Home medications included apixaban, digoxin, atorvastatin, nebivolol, perindopril, moxonidine, and esomeprazole. There was no previous smoking, alcohol, or illicit drug use. According to her relatives, she was last seen well 3 h earlier. She had presented no fever, cough, dyspnea, or preceding illness. At ED presentation, her blood pressure was 83/47 mmHg; heart rate, 90 beats per minute; respiratory rate, 12 breaths per minute; oxygen saturation, 100% on high flow oxygen via a non-rebreather mask; and temperature, 35°C. An arterial blood gas indicated a type 1 respiratory failure (Table 1). On examination, she was completely unresponsive (Glasgow Coma Scale score of 3) with no tonic-clonic activity. Deep tendon reflexes were weak, and Babinski’s sign was bilaterally present. Chest auscultation revealed bibasilar coarse crackles. The remainder of her physical examination was non-contributory. Brain computed tomography (CT) and subsequent CT angiography were normal. CT of the chest and abdomen showed ground glass opacities, predominantly peripheral and involving all five lung lobes, compatible with COVID-19 pneumonia (Fig. 1). Baseline blood analysis was notable for lymphopenia, rhabdomyolysis, crush-related acute kidney injury, and elevated prolactin level (Table 1). A nasopharyngeal swab real-time reverse-transcriptase-polymerase-chain-reaction (rRT-PCR) test was positive for SARS-CoV-2. The blood and urine cultures were negative. Cerebrospinal fluid examination was unremarkable, except for elevated lactate (Table 1). To secure her airway and assure adequate oxygenation, rapid sequence intubation using ketamine 1–2 mg/kg and succinylcholine 1.5 mg/kg was performed and mechanical ventilation started. A bolus of 10 mg midazolam was administered. Norepinephrine titrated up to 0.4 mcg/kg/min was added for refractory hypotension after 30 mL/kg of intravenous crystalloid fluid resuscitation, targeting a mean arterial pressure of 65 mmHg. She received intravenous ceftriaxone 2g and dexamethasone 6mg as recommended for severe COVID-19 pneumonia. As urgent electroencephalography (EEG) is not routinely available at our ED, EEG could not be obtained. With NCSE as presumed etiology of the patient’s coma, she was empirically treated with intravenous valproic acid 40 mg/kg and admitted to the intensive care unit (ICU). The EEG at day 1 showed non-epileptiform generalized background slowing, consistent with antiepileptic drug (AED) treatment. The next 2 days on the ICU, the patient’s clinical condition improved (Glasgow Coma Scale score of 15) due to expected AED responsiveness. At day 4, after being extubated, brain magnetic resonance imaging (MRI) was performed and confirmed NCSE as a cause of her unexplained coma at the ED, showing multifocal bilateral cortical hyperintensities on diffusion weighted images (DWIs) with decreased apparent diffusion coefficient (ADC) and increased signal in T2 and FLAIR with no arterial distribution (Fig. 2). These signal changes, that do not respect vascular territories and are usually reversible over days, typically result from seizure activity, and represent regional vasogenic and cytotoxic edema, reflecting peri-ictal hemodynamic and metabolic changes, respectively. At day 5, she left the ICU and 7 weeks later, she was discharged home from our COVID-19 neurorehabilitation unit, in excellent condition and without neurological deficit. Repeat brain MRI showed complete regression of previously described imaging changes and absence of any type of lesion.

**Table 1:** Patient data at the time of presentation to the Emergency Department

Value (normal range)	
*Venous blood puncture*	
Hemoglobin, g/dL (12.0–16.0)	13.4
White cell count, ×10^9^/L (3.5–11)	8.07
Neutrophil count, × 10^9^/L (1.50–6.70)	6.44
Lymphocyte count, × 10^9^/L (1.20–3.50)	**0.95**
Platelet count, ×10^9^/L (150–440)	202
C-Reactive Protein, mg/L (<5.0)	**56.3**
D-Dimer, ng/mL (0–500)	**7393**
PT, seconds (9.9–11.8)	11.8
APTT, seconds (21.6–28.7)	24
Anti-Factor Xa activity, U anti-Xa/mL	1.06
Sodium, mmol/L (136–145)	**130**
Potassium, mmol/L (3.4–4.4)	3.8
Chloride, mmol/L (98–107)	**89**
Bicarbonate, mmol/L (23–29)	27
Urea, mg/dL (17–48)	**60**
Creatinine, mg/dL (0.50–0.90)	**1.73**
Serum glucose, mg/dL (70–100)	142
Alanine transaminase, U/L (<33)	19
Creatine kinase, U/L (26–192)	**2376**
Myoglobin, mcg/L (<58)	**6063**
Lactate, mmol/L (0.7–2.0)	1.6
Ammonia, mcg/dL (18.7–86.9)	21
Bioactive monomeric prolactin, mcg/L (3.5–18.0)	**29.4**
TSH, mU/L (0.27–4.20)	3.78
Peripheral venous blood culture set (2 independent sets)	Negative
*Arterial blood puncture at ED admission (FiO*_*2*_ *100%)*	
pH (7.35–7.45)	7.47
pCO_2_, mm Hg (32–45)	35
pO_2_, mm Hg (75–104)	234
P/F ratio (>300)	**234**
Saturation O_2_, % (95–98)	100
Carboxyhemoglobin, % (<1.0)	0.9
Methemoglobin, % (<0.5)	0.7
*Arterial blood puncture at ICU admission (FiO*_*2*_ *40%)*	
pH (7.35–7.45)	7.48
pCO_2_, mm Hg (32–45)	35
pO_2_, mm Hg (75–104)	102
P/F ratio (>300)	**255**
Saturation O_2_, % (95–98)	98
*CSF examination*	
CSF macroscopic examination	Crystal clear
CSF opening pressure, cmH_2_0	-
CSF red cell, × 10^9^/L (0–5)	0.1
CSF white cell, × 10^9^/L (0–5)	2.8
CSF glucose, mg/dL (45–80)	75.8
CSF protein, g/L (0.15–0.45)	0.44
CSF oligoclonal bands	-
CSF lactate, mmol/L (1.11–2.44)	**3.76**
CSF PCR for SARS-CoV2	Negative
CSF FilmArray Meningitis/Encephalitis Panel*	Negative
CSF PCR for Herpes Simplex 1	Negative
CSF bacterial and fungal culture	Negative
CSF antinuclear antibodies	Negative
Antibodies against NMDAR, LGi1, CASPR2, GABA-B1/B2, DPPX, AMPA1/2 in serum, and CSF	Negative
Paraneoplastic anti-neuronal antibodies in serum and CSF	Negative
*Urine analysis*	
Leucocytes/μL (<10)	**26**
Erythrocytes/μL (<12)	**26**
Epithelial cells	Absent
Hyalin casts	+++
Crystals	Absent
Bacteria	+
Fungi	Absent
Urine culture set	Negative

*PT* prothrombin time, *APTT* activated partial prothrombin time, *TSH* thyroid stimulating hormone, *CSF* cerebrospinal fluid, *NMDAR N*-methyl-d-aspartate receptor, *LGi1* leucine-rich glioma inactivated 1, *CASPR2* contactin-associated protein 2, *GABA-B* γ-Aminobutyric acid-B receptor, *DPPX* dipeptidyl aminopeptidase-like protein 6, *AMPA1/2* GluR1 and GluR2 subunits of the AMPA receptor; Paraneoplastic antibodies included anti-Hu, anti-Yo, anti-Ri, and anti-amphiphysin

*Escherichia coli K1, *Haemophilus influenzae*, *Listeria monocytogenes*, *Neisseria meningitidis*, *Streptococcus pneumoniae*, *Streptococcus agalactiae*, cytomegalovirus, enterovirus, herpes simplex virus 1 and 2, human herpesvirus 6, human parechovirus, varicella-zoster virus, and *Cryptococcus neoformans*/*Cryptococcus gattii*

## Discussion

Since the onset of the COVID-19 pandemic, a variety of possible neurological manifestations due to SARS-CoV-2 infection have been reported, including, but not limited to, anosmia, dysgeusia, stroke, infectious and autoimmune meningoencephalitis, acute necrotizing encephalopathy, acute disseminated encephalomyelitis, posterior reversible encephalopathy, and Guillain-Barre syndrome [1, 2]. Up to now, only a relatively low number of cases with new onset seizures or status epilepticus in the context of COVID-19 have been published [6–9]. Here, we report a case of NCSE as presenting symptom of a SARS-CoV-2 infection at the ED in a patient with no previous history of epilepsy. Although recently multiple reports of generalized tonic-clonic seizures and one report of focal status epilepticus as initial presentation of COVID-19 at the ED have been described, NCSE as initial presentation of SARS-CoV-2 infection at the ED has been rarely reported [3, 4, 9, 10]. NCSE refers to an electro-clinical state of prolonged non-convulsive seizure activity that manifests primarily as a sudden alteration of consciousness [11]. EEG often—and even more during the COVID-19 pandemic—being unavailable, non-convulsive seizures frequently go unrecognized at the ED; this may have deleterious consequences as NCSE is a critical emergency associated with substantial mortality (25–30%) and irreversible brain damage if not managed in a timely manner [11, 12]. Although pathophysiological mechanisms underlying ictogenesis in SARS-CoV-2 infection remain to be understood. Viral encephalitis and direct invasion of SARS-CoV-2 in the brain may be one of the provoking factors for seizure [9, 13]. In addition, severe COVID-19 disease can result in metabolic and electrolyte imbalances, hypoxia, and cerebrovascular events, all known to facilitate epileptogenesis [9, 13]. SARS-CoV-2 infection also may trigger a “cytokine storm” with rapid release of pro-inflammatory cytokines leading to systemic hyper-inflammation, brain damage, and seizures [13, 14]. In this particular case, with no precipitating factors other than hypoxia-induced facilitation of seizure present, we suggest whether the NCSE in our patient was indeed provoked by the “cytokine storm” associated with COVID-19. It highlights NCSE should not be overlooked in patients with SARS-CoV-2 infection—especially in the elderly—presenting with altered mental status. As mentioned, both delay to diagnosis and longer duration of NCSE are associated with increased morbidity and mortality [11, 12]. Therefore, even in the absence of electroencephalographic evidence to support the diagnosis of NCSE, AED initiation should—in our opinion—not be delayed in clinical suspected NCSE at the ED. Noteworthy, in our patient prompt NCSE recognition and treatment—before any EEG obtained—led to a phenomenal and complete neurological recovery within 48 h. Besides the corresponding clinical picture, transient postictal imaging changes on brain MRI did allow us to retrospectively attribute the definite diagnosis of NCSE in our patient (Fig. 2) [13, 15–17]. This case alerts to the existence of a complete reversible picture of NCSE in SARS-CoV-2 patients and stresses the importance of doing “whatever it takes”—even in the elderly—albeit the clinical situation encountered at the ED initially may appear a perfect storm. Additionally, although NCSE occurs frequently in critical care patients (8–37%), surprisingly few reports mention potential NCSE in COVID-19 ICU patients [12]. Therefore, to conclude, we focus attention on the predisposing context for NCSE in severe COVID-19 at the ICU and speculate that NCSE probably may be considered in certain ICU patients with persistently altered levels of consciousness, agitation, or spatial disorientation—before or after weaning of mechanical ventilation.

## Conclusion

NCSE is a medical emergency, the morbidity, and mortality of which can be decreased by prompt recognition and treatment. NCSE as presenting symptom of COVID-19 at the ED has little been addressed so far. This case report highlights two important issues: first, emergency physicians should keep SARS-CoV-2 infection on their differential diagnosis as more atypical presentations are described, and second, it is important to consider NCSE in COVID-19 patients presenting with altered levels of consciousness at the ED and to not delay appropriate AED treatment. Last but not least, we suggest whether this clinical picture may need more attention at the COVID-19 ICU.

## References

[1] Mao L, Jin H, Wang M, Hu Y, Chen S, He Q, Chang J, Hong C, Zhou Y, Wang D, Miao X, Li Y, Hu B (2020). Neurologic manifestations of hospitalized patients with coronavirus disease 2019 in Wuhan, China. JAMA Neurol.

[2] Pezzini A, Padovani A (2020). Lifting the mask on neurological manifestations of COVID-19. Nat Rev Neurol..

[3] Sokolov E, Hadavi S, Mantoan Ritter L, Brunnhuber F (2020). Non-convulsive status epilepticus: COVID-19 or clozapine induced. BMJ Case Rep..

[4] El Aidaoui K, Ait Benhamou R, Hazim A, Haoudar A, El Kettani C (2021). COVID-19: a potential cause of non-convulsive status epilepticus. Cureus..

[5] Meierkord H, Holtkamp M (2007). Non-convulsive status epilepticus in adults: clinical forms and treatment. Lancet Neurol..

[6] Lu L, Xiong W, Liu D, Liu J, Yang D, Li N, Mu J, Guo J, Li W, Wang G, Gao H, Zhang Y, Lin M, Chen L, Shen S, Zhang H, Sander JW, Luo J, Chen S, Zhou D (2020). New onset acute symptomatic seizure and risk factors in coronavirus disease 2019: a retrospective multicenter study. Epilepsia..

[7] Vohora D, Jain S, Tripathi M, Potschka H (2020). COVID-19 and seizures: is there a link. Epilepsia..

[8] Sharifian-Dorche M, Huot P, Osherov M, Wen D, Saveriano A, Giacomini PS, Antel JP, Mowla A (2020). Neurological complications of coronavirus infection; a comparative review and lessons learned during the COVID-19 pandemic. J Neurol Sci..

[9] Anand P, Al-Faraj A, Sader E (2020). Seizure as the presenting symptom of COVID-19: a retrospective case series. Epilepsy Behav..

[10] Vollono C, Rollo E, Romozzi M, Frisullo G, Servidei S, Borghetti A, Calabresi P (2020). Focal status epilepticus as unique clinical feature of COVID-19: a case report. Seizure..

[11] Sutter R, Semmlack S, Kaplan PW (2016). Nonconvulsive status epilepticus in adults - insights into the invisible. Nat Rev Neurol..

[12] Kinney MO, Craig JJ, Kaplan PW (2018). Non-convulsive status epilepticus: mimics and chameleons. Pract Neurol..

[13] Achar A, Ghosh C (2020). COVID-19-associated neurological disorders: the potential route of CNS invasion and blood-brain relevance. Cells..

[14] Nikbakht F, Mohammadkhanizadeh A, Mohammadi E (2020). How does the COVID-19 cause seizure and epilepsy in patients? The potential mechanisms. Mult Scler Relat Disord.

[15] Adam G, Ferrier M, Patsoura S, Gramada R, Meluchova Z, Cazzola V, Darcourt J, Cognard C, Viguier A, Bonneville F (2018). Magnetic resonance imaging of arterial stroke mimics: a pictorial review. Insights Imaging..

[16] Nakae Y, Kudo Y, Yamamoto R, Dobashi Y, Kawabata Y, Ikeda S, Yokoyama M, Higashiyama Y, Doi H, Johkura K, Tanaka F (2016). Relationship between cortex and pulvinar abnormalities on diffusion-weighted imaging in status epilepticus. J Neurol..

[17] Williams J, Mullins G, Delanty N, Bede P, Doherty CP (2017). The spectrum of peri-ictal MRI changes; four illustrative cases. Seizure..

